# In Situ Controlled Surface Microstructure of 3D Printed Ti Alloy to Promote Its Osteointegration

**DOI:** 10.3390/ma12050815

**Published:** 2019-03-10

**Authors:** Lijun Shan, Abdul Amir H. Kadhum, M.S.H. Al-Furjan, Wenjian Weng, Youping Gong, Kui Cheng, Maoying Zhou, Lingqing Dong, Guojin Chen, Mohd S. Takriff, Abu Bakar Sulong

**Affiliations:** 1 Department of Chemical & Process Engineering, Faculty of Engineering & Built Environment, Universiti Kebangsaan Malaysia, Bangi, Selangor 43600, Malaysia; p93208@siswa.ukm.edu.my (L.S.); amir1719@gmail.com (A.A.H.K.); 2 School of Mechanical Engineering, Hangzhou Dianzi University, Hangzhou 310018, China; gyp@hdu.edu.cn (Y.G.); myzhou@hdu.edu.cn (M.Z.); chenguojin@163.com (G.C.); 3 School of Materials Science and Engineering, State Key Laboratory of Silicon Materials, Zhejiang University, Hangzhou 310027, China; Wengwj@zju.edu.cn (W.W.); Chengkui@zju.edu.cn (K.C.); lingqingdong@zju.edu.cn (L.D.); 4 Research Center for Sustainable Process Technology (CESPRO), Faculty of Engineering and Built Environment, Universiti Kebangsaan Malaysia, Bangi, Selangor 43600, Malaysia; sobritakriff@ukm.edu.my; 5 Department of Mechanical and Materials Engineering, Universiti Kebangsaan Malaysia, Selangor 43600, Malaysia; abubakar@ukm.edu.my

**Keywords:** 3D printing, Ti implants, osteogenesis, micro-nano structured surface, in situ control

## Abstract

It is well known that three-dimensional (3D) printing is an emerging technology used to produce customized implants and surface characteristics of implants, strongly deciding their osseointegration ability. In this study, Ti alloy microspheres were printed under selected rational printing parameters in order to tailor the surface micro-characteristics of the printed implants during additive manufacturing by an in situ, controlled way. The laser path and hatching space were responsible for the appearance of the stripy structure (S), while the bulbous structure (B) and bulbous–stripy composite surface (BS) were determined by contour scanning. A nano-sized structure could be superposed by hydrothermal treatment. The cytocompatibility was evaluated by culturing Mouse calvaria-derived preosteoblastic cells (MC3T3-E1). The results showed that three typical microstructured surfaces, S, B, and BS, could be achieved by varying the 3D printing parameters. Moreover, the osteogenic differentiation potential of the S, B, and BS surfaces could be significantly enhanced, and the addition of nano-sized structures could be further improved. The BS surface with nano-sized structure demonstrated the optimum osteogenic differentiation potential. The present research demonstrated an in situ, controlled way to tailor and optimize the surface structures in micro-size during the 3D printing process for an implant with higher osseointegration ability.

## 1. Introduction

Three-dimensional (3D) printing technology is one of the most effective and economical ways to make individualized implants [1] because of the ability to structure arbitrary and complicated geometry with low cost and short time [2]. Titanium (Ti) and its alloy have good mechanical properties, strong corrosion resistance, high mechanical properties, and excellent cytocompatibility. Therefore, they have been widely used for orthopedic surgery and other medical applications [3,4,5,6,7,8,9,10]. There has been plenty of research about 3D-printed Ti implants. Nonetheless, most of this research was focused on the mechanical properties instead of the surface structures of the implants [11,12,13]. However, the surface structures of implants are vital to regulate cell viability in the development of tissue engineering and regenerative medicine [14,15]. The microstructure of metal implants plays a vital role in cellular biocompatibility [16,17]. On the one hand, microstructures are conducive to cell adhesion and proliferation; therefore, they can significantly enhance cell responses [18,19]. On the other hand, microstructures are able to determine the mechanical interlocking degree with the host bone and promote complex interaction between a synthetic foreign body and the host bone, which will further affect the key property of an implant’s osseointegration ability and determine the success or failure of the implants. In addition, researchers have demonstrated that unique nanostructures may trigger related signal channels of cells [20,21], further affecting the pivotal role of cell morphology, adhesion, proliferation, and migration [22]. Thus, the nanostructure of metal implants will make a difference in cellular response and determine the bone-bonding strength with the host bone. It has been indicated that the micrometer and nanometer scale of surface roughness could improve the adhesion and growth of cells on a biomaterial surface [23,24,25].

At present, to promote the biological functions of 3D printed implants, surface modification, such as biomimetic coating, Hydroxyapatite (HAP) polymerization, acid etching, sandblasting, heat treatment, chemical treatment, Mg ion implantation, and so on [13,26,27,28,29,30], is applied to form microstructures on the surface of 3D-printed implants. Nevertheless, surface modification is time consuming and expensive. To construct specific microstructures during the 3D printing process will work wonders in promoting the osseointegration ability of the 3D-printed implant; however, how to control the surface microstructure in situ remains a challenge.

During the 3D printing process, microstructures are able to be developed directly when the Ti alloy microsphere is melted by the laser. Different printing parameters, such as laser path and contour printing, will cause a significant change in the microstructure of the 3D-printed implants [31]. A bi-directional laser path will make stripy structures on both the top and side surface, while contour rescan will flattening the stripy structures on the side surface [32] but form a spherical topography. A composite structure of stripy and spherical characteristics can be developed without contour rescanning. Meanwhile, hatching space and laser powder may regulate the surface microstructure as well. These parameters are an efficient way to control the surface structures in situ, which could further regulate the cell behavior and viability [16,17,33].

In this study, we attempted to adopt an in situ, controlled way to control surface micro-characteristics of titanium alloy implants in the period of their additive manufacturing by selecting rational printing parameters. The resulting, typical, surface, micro-sized morphologies were evaluated with respect to cytocompatibility, and the relationship between the tailored morphologies and cellular osteogenic differentiation was also investigated with the aim to provide guidance for 3D-printed implants with better osseointegration.

## 2. Materials and Methods

### 2.1. 3D Printing of Ti Alloy Implants

A 3D printing machine (EP-M250, Shining 3D Co. Ltd., Hangzhou, China) with a fiber laser was used to construct the Ti alloy implants. The 3D printing process parameters (laser power, scanning rate, hatching space, and layer thickness) were controlled and adjusted, specifying the laser power to ±30% of 500 W. The hatching space was ±0.03 mm of 0.08 mm, the scanning rate was ±30% of 1280 m·s^−1^, and the layer thickness was 0.03 mm. The printed Ti alloy implants (TiP) were constructed in the form of square substrates (250 × 250 × 300 mm^3^) in the presence of an Argon atmosphere (~500 ppm O_2_). The constructing process started by laying a thin, even layer of Ti6Al4V powder on a substrate plate in a building chamber. After the Ti powder was laid, a high-power focused laser (DLR-976-1500 IPG Photonics, Oxford, MA, USA) was used to melt the selected areas pursuant to the processed data. Once the laser scanning was finished, the constructing platform was lowered and a new layer of Ti powder was deposited on top, where the laser proceeded to scan a new layer. The process was repeated for successive layers of Ti powder until reaching the required thickness (1 cm). Once the 3D printing process was finished, wire cutting was used to cut the printed Ti samples from the sample substrate plate. 

### 2.2. Hydrothermal Treatment

Hydrothermal treatment was used to obtain the nanostructure on the 3D-printed, Ti alloy, microsphere surface. The hydrothermal solution was prepared from 5 M NaOH. 80 mL hydrothermal solution, and the 3D-printed Ti alloy substrates (TiP) were placed into a 100 mL Teflon vessel of the hydrothermal autoclave (YZHR-100, Yan Zheng Instrument, Shanghai, China). The hydrothermal autoclave was put into an oven at 80 °C for 24 h. The autoclave vessel was then taken out and cooled down to room temperature. TiP substrates with nano-microstructures were taken and washed with deionized water and ethanol. 

### 2.3. Characterization of Prepared Ti Alloy Implants

Field-emission scanning electron microscope (FESEM, Hitachi Su-70, Hitachi, Tokyo, Japan) was used to characterize the surface topography of the prepared implants. Furthermore, It was used to observe the cell adhesion on different samples.

### 2.4. Protein Adsorption

The protein adsorption was studied by soaking the samples in a 24-well plate with a 500 μL solution of minimum essential medium-alpha modification (α-MEM) containing 10% fetal bovine serum (FBS) at 37 °C for 24 h. To detach the protein, the samples were placed in another 24-well plate and put in 500 μL of 1% sodium dodecyl sulfate solution (SDS-CH_3_(CH_2_)_11_SO_4_Na, Sinopharm Chemical Reagent Co. Ltd., Shanghai, China) for 2 h. The bicinchoninic acid assay kit was utilized to determine the detached protein concentration in the solution. 

### 2.5. Cell Culture

Mouse calvaria-derived preosteoblastic cells (MC3T3-E1, CRL-2594, and ATCC) were employed. The MC3T3-E1 cells were cultured in minimum essential medium-alpha modification (α-MEM, Gibco) supplemented with 10% FBS (PAA, Australia), 1% antibiotic solution containing 10,000 units/mL penicillin and 10 mg/mL streptomycin (Gibco), 1% sodium pyruvate (Gibco), and 1% MEM nonessential amino acids (Gibco). The cells were cultured under a humidified 5.0% CO_2_ atmosphere at 37 °C. The cultured cells were trypsinized with 0.25% trypsin and 1 mM ethylenediaminetetraacetic acid (EDTA, Gibco), centrifuged, spread in fresh culture medium, and subcultured on substrate plants.

### 2.6. Cell Viability

CCK-8 (Dojindo Laboratories, Kumamoto, Japan) assay was applied to evaluate cell viability after a density of 5 × 10^4^ cells/cm^2^ cell was cultured over 1 day and 5 days. The samples were washed 3 times by PBS, 500 μL cell suspension was added with a 50 μL CCK-8 solution in new 24-well plates, incubated for 3 h at 37 °C, and then, 120 μL mixed solution was transferred to a 96-well plate. The absorbance value (OD) was tested by a microplate reader at 450 nm.

### 2.7. Cell Differentiation

Cell differentiation was evaluated by alkaline phosphatase (ALP) activity. MC3T3-E1 cells were inoculated on different samples in a 24-well plate. The culture medium was changed every 3 days. After 7 or 14 days of culture, the samples were transferred to another 24-well plate. The cells were lysed for 10 min and then centrifuged for 15 min. In order to obtain the content of ρ-nitrophenyl phosphatase, 20 μL supernate was used by ALP assay kit (Wako, Osaka, Japan) and then determined by a microplate reader (the absorption wavelength was set to 405 nm). Meanwhile the content of the cellular proteins was determined through a Bicinchoninic acid (BCA, Thermo Fisher Scientific, Shanghai, China) kit by using 25 μL supernate. ALP activity was the value of *ρ*-nitrophenyl phosphatase divided by the content of the cellular proteins. 

### 2.8. Cellular Cytoskeleton Morphology

Cellular cytoskeleton morphology was observed by laser scanning confocal microscopy (A1, Nikon, Tokyo, Japan) after immunofluorescent staining. Cells with a density of 1 × 10^4^ cells/well were cultured for 1 day on the 3D-printed Ti substrate and fixed for 15 min in 4% paraformaldehyde. Then, the cells were permeabilized in 0.4% Triton X-100/PBS for 15 min. A quantity of 250 μL phalloidin (PHDR1, Cytoskeleton, Inc.) was used to stain the actin of the cell cytoskeleton for 30 min. Then, the samples were washed by 0.05% Tween 20/PBS 2 times. Next, 250 μL DAPI (4’,6-diamidino-2-phenylindole)(DAPI, H-1200, VECTOR) was applied to stain the nucleus. Then, 0.05% Tween 20/PBS was used to wash the samples 2 times, and antifade mounting medium was added and placed without light and ultimately observed by laser scanning confocal microscopy (A1, Nikon).

### 2.9. Statistical Analysis

A mean of ±standard deviation (SD) was applied for all the data mentioned. By using one-way analysis of variance (ANOVA), statistical analyses were carried out and Scheffe’s post hoc test with SPSS software was used for multiple comparison tests. It was considered statistically significant when * *P* < 0.05 (* *P* < 0.05, ** *P* < 0.01, *** *P* < 0.001).

## 3. Results

### 3.1. The In Situ Tailored Surface Structures of 3D-Printed Ti Alloy Implants

**Figure 1:**
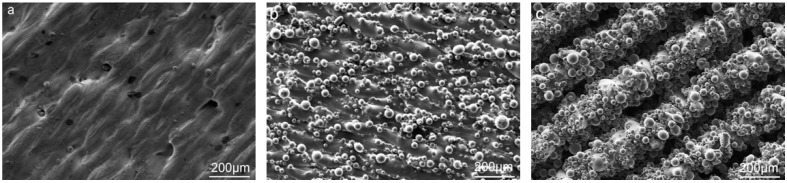

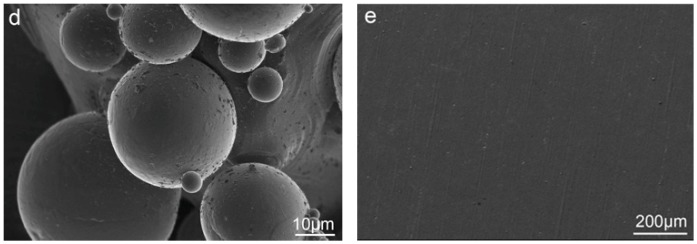
SEM images of three typical surface topographies: (**a**) stripy structure (S), (**b**) bulbous structure (B), and (**c**) bulbous–stripy composite surface (BS); (**d**) high-resolution images of BS microstructures; and (**e**) topography of Ti substrate (control).

There were three typical microstructures tailored by varying the printing parameters. A stripy structure (s) was constructed by zig-zag laser path, a bulbous structure (B) was formed with contour scanning, and the bulbous–stripy composite structure (BS) was obtained without contour scanning. The surface topography of the 3D-printed Ti alloy implants were characterized by scanning electron microscopy [34], as shown in Figure 1.

The results showed that the surface structure of S was striation, and the width of the striation was 90 μm. The amount of titanium microsphere on the surface of the Ti alloy was estimated to be ~60 per mm^2^ (Figure 1a); however, the surface of B was denser than the surface S of microspheres of various sizes, ranging from 10 to 53 μm. The amount of microsphere on the surface of the Ti alloy was estimated to be ~1000 per mm^2^. As shown in Figure 1c, the surface structure of BS was a combination of striation and microspheres. There were plenty of microsphere structures arranged on line-like structures. The width of the line-like structure was an estimated ~160 μm, and the number of microsphere structures was estimated ~700 per mm^2^. This distinctive microstructure feature was comparable in size to the cells and thus beneficial to cell response.

Higher resolution images revealed that the microspheres’ structures were melted into the surface and formed a firm and rough microstructure compared with commercial Ti, which is smooth, as shown in Figure 1d,e.

### 3.2. The Post Hydrothermal Treatment of the Tailored Surface Structures

**Figure 2:**
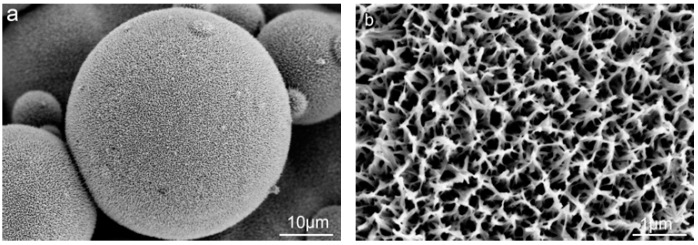
(**a**) SEM image of microstructures after hydrothermal treatment and (**b**) high-resolution images of porous nanostructures on the microstructure.

Hydrothermal treatment was carried out to introduce TiO_2_ nanostructures (Figure 2). The SEM images illustrate nano-pores on the surface of the microsphere with an estimated average size of ~200 nm, while maintaining the microstructure feature topography.

### 3.3. Surface Composition

**Figure 3:**
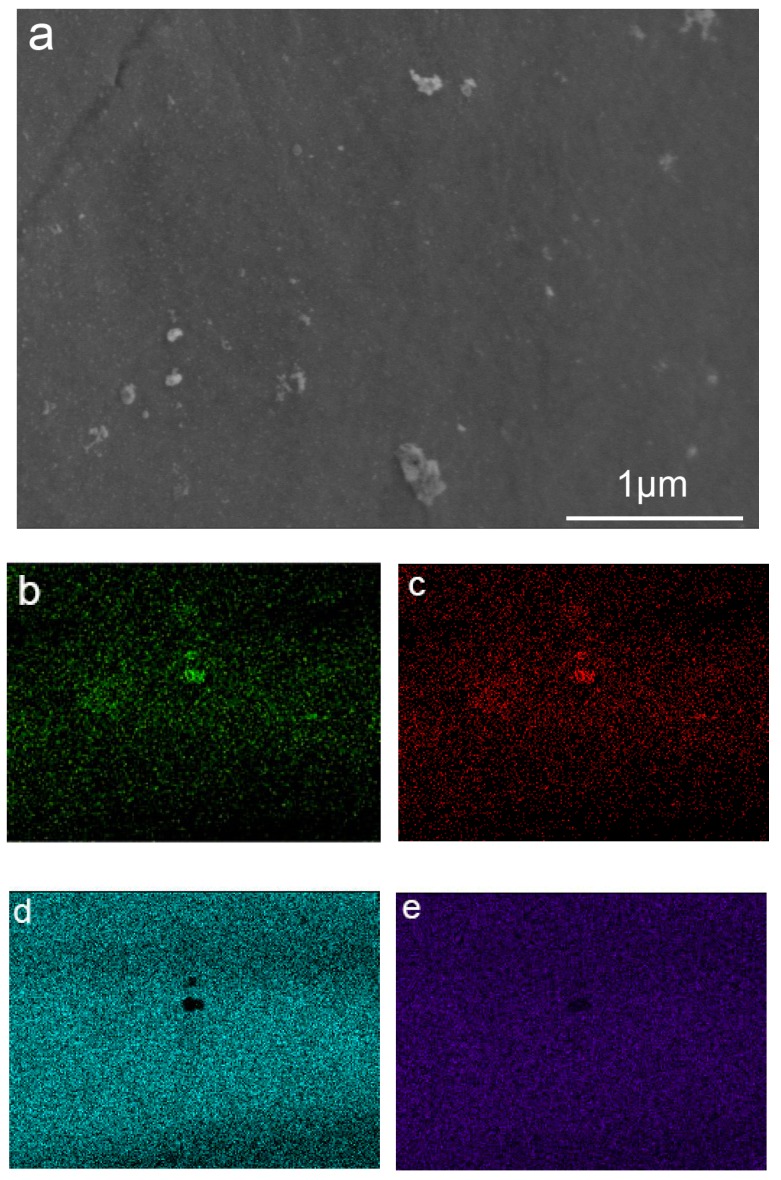
SEM image and element mappings of precipitates on the surface of the 3D printed samples (**a**,**b**) O Kal; (**c**) Al Kal; (**d**) Ti Kal; (**e**) V Kal.

Observed by SEM, the surface of T, B, and BS are not completely smooth. There is graininess on the surfaces, as shown in Figure 3a. To identify the graininess, Energy Dispersive Spectrometer (EDS) was applied, as shown in Table 1. The titanium, aluminum, and vanadium elements on the microsphere and substrate were similar and were speculated to be Ti6Al4V. The oxygen content on the microsphere was higher due to oxidization during the heat treatment. The elements of graininess had a much higher quantity of aluminum and oxygen. Meanwhile, surface scanning by SEM was used to analyze the elements of the precipitates. As shown in Figure 3b–e, aluminum and oxygen elements were gathering on the graininess. The results of the two items certified that the graininess was Al_2_O_3_.

### 3.4. In-Vitro Evaluation

#### 3.4.1. Protein Adsorption

**Figure 4:**
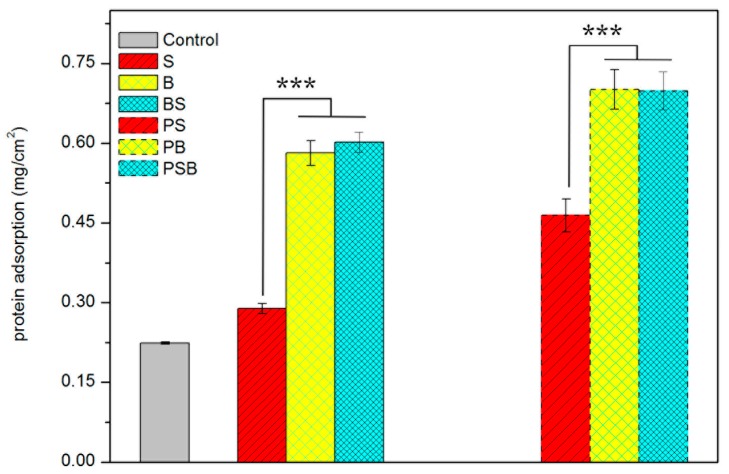
Protein adsorption ability analysis of MC3T3-E1 cells on different surface structures.

The protein adsorption properties of different samples were tested, as shown in Figure 4. N and BS had similar amounts of protein adsorption quantities but much more than the control and S. Thus, microspheres played a vital role in protein adsorption. After hydrothermal treatment, the samples had higher protein adsorption simultaneously, and furthermore, defined nanostructures could improve protein adsorption.

#### 3.4.2. Cell Viability

**Figure 5:**
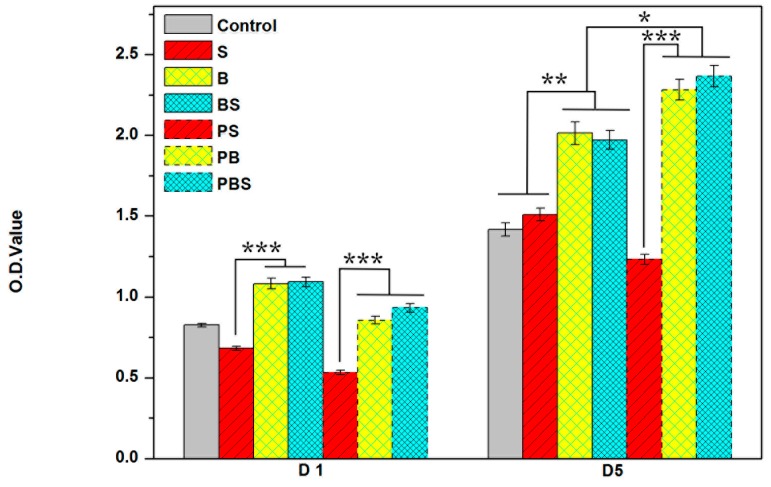
Cellular viability evaluation (CCK-8 assay) of MC3T3-E1 cells on different surface surfaces after one-day and five-day cultures.

After a one-day culture, the MC3T3-E1 cells’ adhesion ability was investigated by a CCK-8 assay analysis. As shown in Figure 5, B and BS had similar cell attachment ability, which proves that spherical structures had a great impact on cell attachment. However, the porous structures had lower cell attachment.

Cell proliferation was tested after a five-day culture. As shown in Figure 5, B and BS exhibited more markedly increased cell proliferation than the control and S, again verifying that spherical microstructures were able to motivate cell viability. The cell proliferation of the nano-microstructures of B and BS were improved, while S was decreased. These data illustrated that the nanostructure could play a positive role with the help of microspherical structures.

#### 3.4.3. Cell Distribution on Various Surfaces

**Figure 6:**
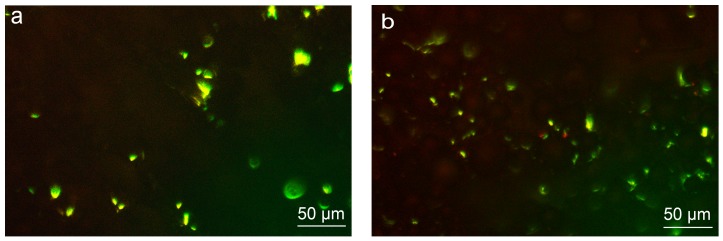

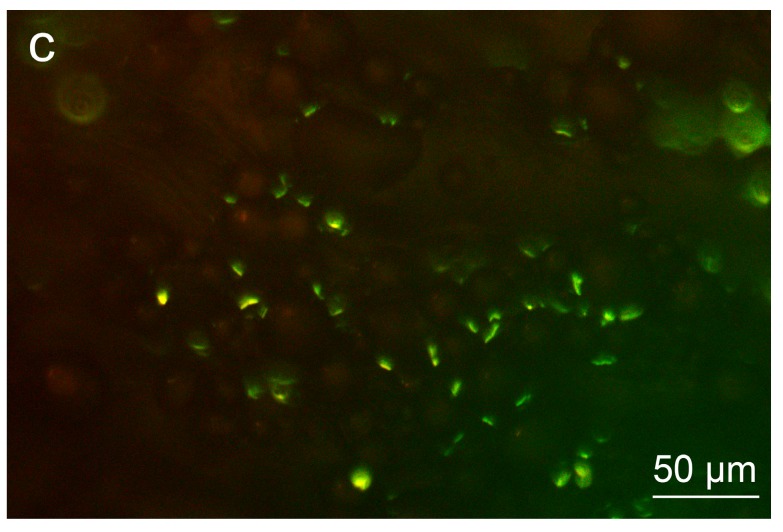
The fluorescence staining images representing the distribution of cells (**a**) S, (**b**) B, (**c**) BS.

As shown in Figure 6, the cells were distributed on the porous structure of S (Figure 6a). However, when observing B (Figure 6b) and BS (Figure 6c), the cells were unable to crawl onto the microspheres, only distributing around the microspheres. This indicated that microstructures play an important role in cell distribution. 

**Figure 7:**
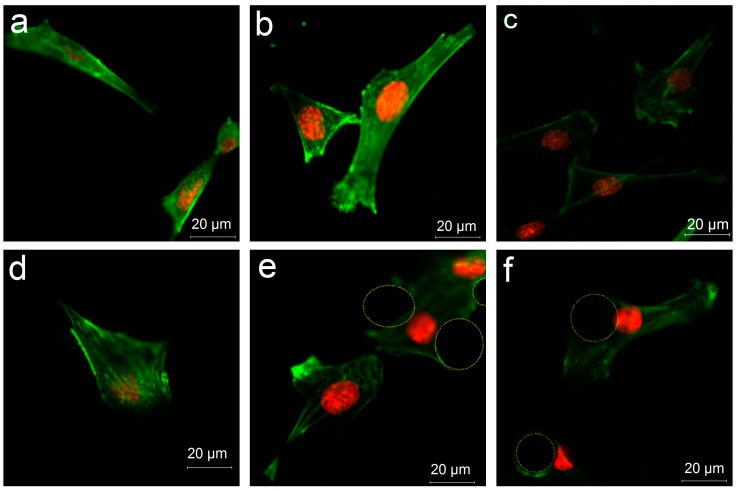
The confocal laser scanning microscopy (CLSM) images representing cellular distribution on different surfaces: (**a**) S, (**b**) B, (**c**) BS, (**d**) PS, (**e**) PB, (**f**) PBS. The circles identify the nanostructured microspheres.

To further confirm how the surface structures affect the distribution of cells, cell staining was applied. Confocal laser scanning microscope (CLSM) images showed that on B (Figure 7b) and BS (Figure 7c), the cells were distributed on the substrate instead of on the microspheres. However, cells were able to attach on and around the microsphere after nanostructures were introduced, as shown in Figure 7e–f. Thus, nanostructures were conductive to cell adhesion.

#### 3.4.4. Cell Differentiation

**Figure 8:**
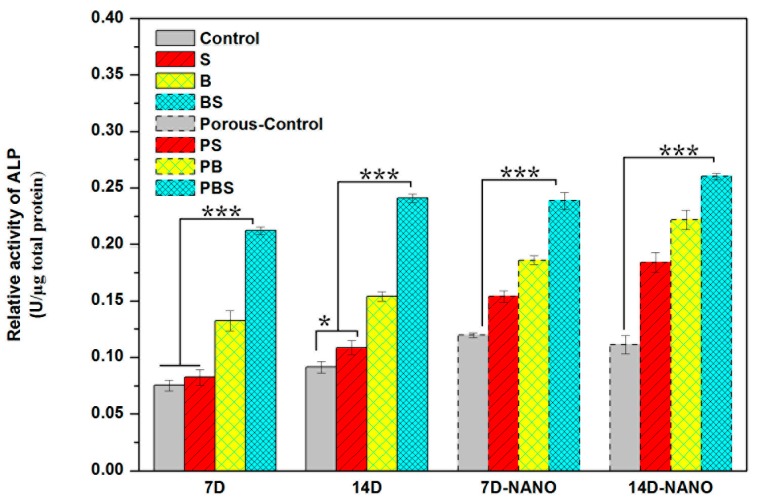
Alkaline phosphatase (ALP) expression of MC3T3-E1 cells on different surface structures after seven-day and 14-day cultures.

Alkaline phosphatase (ALP) expression of MC3T3-E1 cells as an osteogenic differentiation indicator in the early stages is shown in Figure 8. The expression of ALP protein on (BS) was remarkably enhanced at day 7 and 14. The cells on BS showed significant upregulated ALP activity compared with that of the other structures. In addition, BS showed much more differentiation at day 14, notably higher than the other structured surfaces. The variability in cell differentiation for the structures revealed a similar trend with respect to cell adhesion, as well as proliferation. Obviously, the cells on the BS structure had better osteogenic differentiation, which might be attributed to the initial cell viability.

## 4. Discussion

### 4.1. In Situ, Controlled Surface Structure

#### 4.1.1. In Situ, Controlled Surface Microstructure

**Figure 9:**
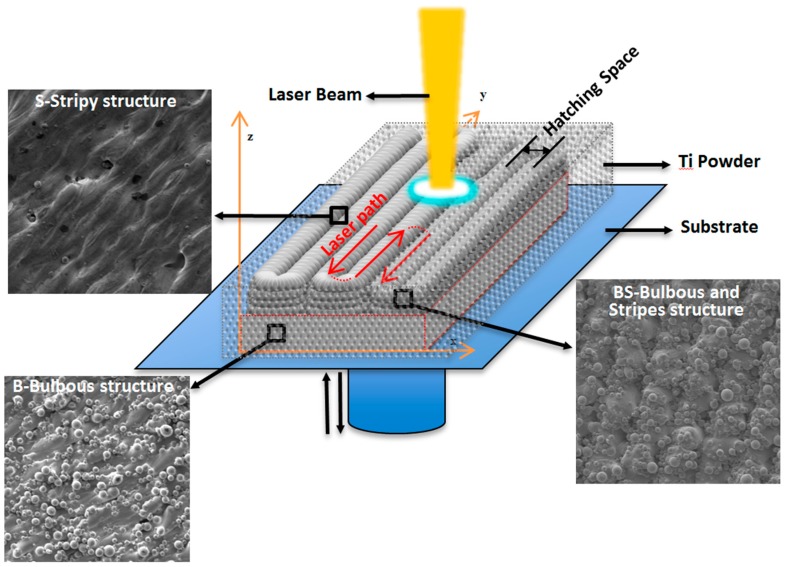
Schematic of the formation of three typical surface structures by regulating the 3D printing parameters.

During the 3D printing process, Ti alloy powders are laid to form a layer with a set thickness, and then, a laser beam scans the powder selectively with a given laser power, hatching space, and scanning path. The powders, upon scanning, are melted immediately and then rapidly cooled and solidified. The surrounding powder that has not been scanned or melted is adsorbed by the melted liquid Ti alloy due to capillary force and then firmly attached when the liquid Ti alloy solidifies. At the end of a layer printing, the contour is rescanned if needed. Once the layer is completely printed, powders are laid on the previous layer and melted again, the above operations are repeated, and the layers are superimposed above layers until the printing is completed. The schematic of these Selective Laser Melting (SLM) processing parameters are shown in Figure 9.

Compared with forming different surface structures by controlling printing angles, such as a 0 angle, 45 angle, and 60 angle [35], varying the printing parameters is a much more efficient and easier way to tailor surface structure.

When the laser scans the powder in bi-directional (zig-zag) path, the powder is melted by a high-power laser along the laser path and the surface temperature difference between the solidified part and the laser beam lead to surface tension force exerting a shear force on the liquid surface, thus causing a rippling effect and forming a stripy structure (named as S) along laser path. The width of the strip will be determined by the hatching space when the hatching space increased. 

When the laser beam reaches the edge of the samples, the laser path is retraced, and it results in the formation of embossment on the side surface of the sample. After a few layers of superimposing the embossment, a stripy structure along the Z-axis formed, with double the width of the hatching space. Meanwhile, as a result of capillary force, the surrounding powder that has not been scanned or melted is firmly adsorbed on the side surface of the sample by a molten pool when the molten material is cooled and solidified, forming a spherical structure on the stripy structures. Thus, a composite structure of stripy structures and bulbous structures was developed on the side face of the sample, called BS (bulbous–stripy structure).

When the laser rescans the contour of the samples, the stripy structure along the Z-axis is melted under a high-energy laser beam, the molten pool is flattened, and wettability is increased. The stripy structures then disappear, and the surrounding powder that has not been scanned and melted is adsorbed and spherized, resulting in a bulbous microstructure (called B). 

Hence, during the 3D printing process, by adjusting the hatching space, laser path, and contour rescanning, the topography of the samples can be tailored in situ. In comparison with sandblasting and other post-treatments, an in situ, controlled way of altering the surface microstructure is much more convenient and efficient [29,36]. Furthermore, this method is able to avert radial fracture or damage to the implants caused by post-treatment [37].

#### 4.1.2. Construction of Nanostructure

Hydrothermal treatment was carried out under high temperature and high pressure. The chemical bond of O-Ti-O was broken by the NaOH solution. Na^+^ ions penetrated into the TiO_2_ crystal, leading to the instability of the crystal and peeling off from the sample surface, becoming flake-like sodium titanate, after which it dissolved and diffused in the solution. Sodium titanate could not immediately diffuse into the solution very evenly; therefore, it concentrated on the surface of the sample and led to the concentration difference of sodium titanate, promoted the growth of sodium titanate in multi-dimensional scaling, and formed a porous nanostructure on the surface.

### 4.2. Analysis of Cellular Responses on the Tailored Surface Structures of the Implants

Surface topography formed by microscale and nanoscale structures could make a difference with respect to the cell proliferation, behavior, and differentiation and further affect the integration between the implants and bone tissue [33,38,39,40]. In this study, three different surface topographies, S, B, and BS, were tailored in situ on implants by 3D printing (Figure 1). Furthermore, hydrothermal treatment was applied to form nanostructures (Figure 2). Cell distribution on different surfaces was observed. The cells could rarely distribute on the microsphere substrate (Figure 6 and Figure 7a–c). After treatment, the cells were capable of attaching on and around the microspheres (Figure 7d–f). 

The cells were not able to crawl onto the microstructures, which were larger, but a porous nanostructure provided more adhesion sites for the cells. This may be caused by the improved protein absorption by the nanostructured surfaces, as Woo noted that nanostructures could create a more favorable environment for cells and improve the features of protein adsorption [41]. 

Cell adhesion is one of the first factors causing implants to adhere with the host bone and further influencing cell behavior, such as cell proliferation and differentiation. High cell proliferation could lay the foundation for cell differentiation. B and BS had much higher cell attachment ability and cell differentiation ability than S and the control (Figure 5). This demonstrated that surface topography plays a vital role in cell viability, and microspheres could dramatically motivate cell adhesion and proliferation. It is noteworthy that the nanostructured group had lower cell adhesion ability but higher cell proliferation ability than the untreated group. As a result of the introduction of Al_2_O_3_, precipitation was detrimental to cell adhesion (Figure 3) but beneficial to cell proliferation. Moreover, cell proliferation on different structures illustrated that the nanostructure could play a positive role with the help of a microspherical structure.

Cell osteogenic differentiation is considered the foremost property for implants, because it is closely related with osseointegration ability. It has been demonstrated that rough surface microstructures may hasten the osseointegration of implants [42]. BS showed favorable cell differentiation ability, which was notably higher than other structured surfaces (Figure 8). Cell differentiation ability displayed the same trend as protein adsorption (Figure 4) and cell viability (Figure 5). Hence, it can be concluded that surface topography had an impact on protein adsorption and then made a difference with respect to the cell adhesion site, further affecting cell viability and differentiation. Thus, osseointegration of implants can be regulated by tailoring surface structure.

## 5. Conclusions

In this work, we demonstrated that tailored surface microstructures of 3D-printed implants could be regulated in situ by changing printing parameters. The tailored surface microstructures played a crucial role in the biocompatibility of the implants. Further, the initial osteointegration improved when the porous nanostructures were formed by hydrothermal treatment. This work provides theoretical guidance and technical support for in situ surface design and processing of 3D-printed implants with high osteogenic and biological responsiveness.

## Figures and Tables

**Table 1:** EDS determination of the element content of the different areas of the three-dimensional (3D)-printed surface.

Area	Ti K	Al K	V K	O K
Substrate	73.98	5.51	3.28	14.86
Microsphere	72.44	5.46	3.16	15.87
Graininess	35.22	16.00	2.01	46.77
